# Platelet membrane-camouflaged silver metal-organic framework drug system against infections caused by methicillin-resistant *Staphylococcus aureus*

**DOI:** 10.1186/s12951-021-00978-2

**Published:** 2021-08-04

**Authors:** Rong Huang, Guang-Qing Cai, Jian Li, Xi-Sheng Li, Hai-Ting Liu, Xue-Ling Shang, Jian-Dang Zhou, Xin-Min Nie, Rong Gui

**Affiliations:** 1 Department of Blood Transfusion, The Third Xiangya Hospital, Central South University, Hunan Changsha, China; 2 Department of Laboratory Medicine, The Third Xiangya Hospital, Central South University, Hunan Changsha, China; 3 Department of Orthopedics, Changsha Hospital of Traditional Chinese Medicine, Changsha Eighth Hospital, Hunan Changsha, China

**Keywords:** Platelet, Metal-organic framework, Vancomycin, Methicillin-resistant, *Staphylococcus aureus*

## Abstract

**Background:**

Due to the intelligent survival strategy and self-preservation of methicillin-resistant *Staphylococcus aureus* (MRSA), many antibiotics are ineffective in treating MRSA infections. Nano-drug delivery systems have emerged as a new method to overcome this barrier. The aim of this study was to construct a novel nano-drug delivery system for the treatment of MRSA infection, and to evaluate the therapeutic effect and biotoxicity of this system. We prepared a nano silver metal-organic framework using 2-methylimidazole as ligand and silver nitrate as ion provider. Vancomycin (Vanc) was loaded with Ag-MOF, and nano-sized platelet vesicles were prepared to encapsulate Ag-MOF-Vanc, thus forming the novel platelet membrane-camouflaged nanoparticles PLT@Ag-MOF-Vanc.

**Results:**

The synthesized Ag-MOF particles had uniform size and shape of radiating corona. The mean nanoparticle size and zeta potential of PLT@Ag-MOF-Vanc were 148 nm and − 25.6 mV, respectively. The encapsulation efficiency (EE) and loading efficiency (LE) of vancomycin were 81.0 and 64.7 %, respectively. PLT@Ag-MOF-Vanc was shown to be a pH-responsive nano-drug delivery system with good biocompatibility. Ag-MOF had a good inhibitory effect on the growth of three common clinical strains (*Escherichia coli*, *Pseudomonas aeruginosa*, and *S. aureus*). PLT@Ag-MOF-Vanc showed better antibacterial activity against common clinical strains in vitro than free vancomycin. PLT@Ag-MOF-Vanc killed MRSA through multiple approaches, including interfering with the metabolism of bacteria, catalyzing reactive oxygen species production, destroying the integrity of cell membrane, and inhibiting biofilm formation. Due to the encapsulation of the platelet membrane, PLT@Ag-MOF-Vanc can bind to the surface of the MRSA bacteria and the sites of MRSA infection. PLT@Ag-MOF-Vanc had a good anti-infective effect in mouse MRSA pneumonia model, which was significantly superior to free vancomycin, and has no obvious toxicity.

**Conclusions:**

PLT@Ag-MOF-Vanc is a novel effective targeted drug delivery system, which is expected to be used safely in anti-infective therapy of MRSA.

**Graphic abstract:**

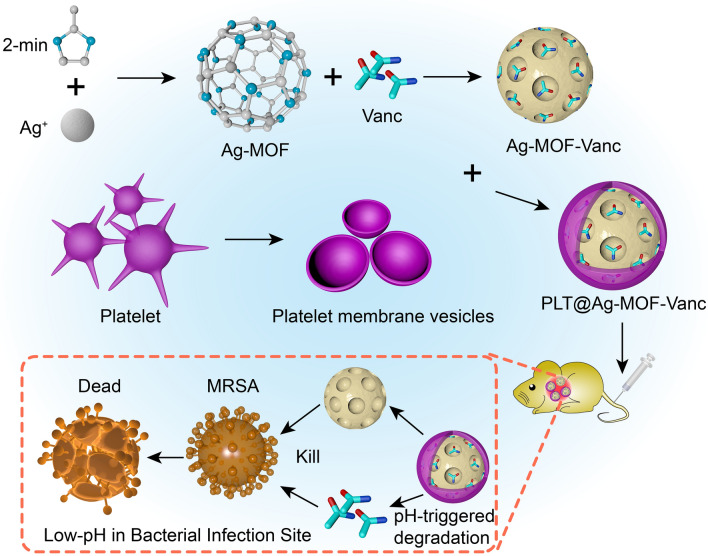

## Introduction

*Staphylococcus aureus *(*S. aureus*) is a kind of gram-positive bacteria with strong pathogenicity. It can cause infections of the skin and soft tissues, and internal organs; it is also the most common gram-positive bacterial species that causes sepsis [1]. More severe cases may progress to multiple organ failure, diffuse intravascular coagulation, lactic acidosis, and even death. Antibiotics are the first-line drugs in bacterial infection treatment; however, due to the abuse of antibiotics and long-term natural selection of bacteria, drug-resistant bacteria appeared, posing a major threat to the existing antibiotics in clinical practice [2]. At present, more than 90 % of *S. aureus* clinical isolates are resistant to penicillin [3]. To overcome penicillin resistance, scientists developed methicillin, but within two years of using methicillin, in 1961, Jevons found methicillin-resistant *Staphylococcus aureus* (MRSA) for the first time in the UK [4]. MRSA strains were resistant not only to methicillin, but to all penicillins, cephalosporins, and carbapenems [5], making MRSA more harmful than those bacteria that were resistant to “only” one kind of antibiotic. In addition, MRSA strains have become resistant to more and more other antibiotics, such as fluoroquinolones, macrolides, aminoglycosides, and clindamycin [6–9]. According to the US Centers for Disease Control and Prevention (CDC), MRSA has been considered as a serious threat after taking into account various factors, such as treatability, mortality, burden on the healthcare-infrastructure and the community, prevalence and increasing trends of resistance [10]. MRSA remains one of the difficult-to-treat ESKAPE pathogens (*Enterococcus faecium*, *S. aureus*, *Klebsiella pneumoniae*, *Acinetobacter baumannii*, *Pseudomonas aeruginosa*, and *Enterobacter* species), which is the most serious threat to the development of untreatable multidrug-resistant (MDR) infections [11, 12].

MRSA has developed multiple resistance mechanisms, including the thickened bacterial cell wall, the increased efflux pumps on cell membrane, the drug targets mutations, drugs modifications and the altered bacterial colonization state [3, 13–15]. Thus, clinical treatment of MRSA infection is a challenging problem. The main drawbacks of current antibiotics are associated side effects and increasing resistance. Although vancomycin remains the first-line therapy of choice, it has some limitations, such as high doses, slow antimicrobial rate, nephrotoxicity, low tissue penetration, and low oral absorption [16–18]. Rising resistance has also shaken faith in vancomycin [19, 20]. In addition to vancomycin, daptomycin is an alternative treatment which can circumvent resistance that occurs in MRSA treatment [21, 22]. Daptomycin may be used for MRSA bacteremia, but not for pneumonia of lower respiratory, and is associated with eosinophilic pneumonia and rhabdomyolysis [23, 24]. Furthermore, emergence of resistance to daptomycin during therapy was a well-described phenomenon that threatens the clinical use of this antibiotic, further limiting the therapeutic options against MRSA [25, 26]. Combination antibiotic therapy is an under-explored new hope but not always effective [22, 27]. Moreover, MRSA antibiotic treatments have also been shown to be non-specific, as they kill beneficial human bacteria, leading to dysbiosis (microbial imbalance) [28].

Current antibiotics against MRSA have either failed or shown severe side effects, making it necessary to find alternative strategies in addition to the discovery of novel drug and the reuse of current drugs. Using nanomaterial approach to combat the antibiotic resistance could offer new opportunities to address these challenges [29]. Nanomaterials have unique advantages including small particle size, high surface area, high drug carrying capacity and targeting ability, which can be used for advanced delivery of antibiotics or other antibacterial drugs. Nanomaterials could effectively improve the permeability of the cell membrane for loaded drugs, enhance intracellular drug accumulation, improve the antibacterial activity of antibiotics against drug-resistant strains, provide a variety of bactericidal mechanisms, and inhibit the formation of *S. aureus* biofilm [30].

Some metal-based nanoparticles have natural antimicrobial activity and therefore show great potential for antimicrobial therapy [31]. Silver is one of the most effective and commonly used antibacterial materials, which can interfere with key functions in antimicrobial resistant microorganisms [32, 33]. Silver breaks down the cell wall by interacting with the proteins of cell wall, inhibits cell division by interacting with DNA and RNA, interferes with signal transduction, and leads to the production of ROS [34, 35]. In view of antibiotics overuse leading to the increase of drug-resistant strains, silver nanoparticles (AgNPs) have emerged as a potential antibacterial agent. Combined use of AgNPs with antibiotics is a known strategy to overcome multiple drug resistance, which can enhance the overall antibacterial activiy [18, 36, 37]. It also allows to reduce the dosages of the antibiotics and attenuate side effects [38, 39]. Metal-organic frameworks (MOFs) are polymers assembled by metal ions and organic ligands through coordination. Coordination polymers contain many metal ions and ligands and they have flexible structure and unique properties; moreover, they are organic–inorganic hybrid materials with high porosity and specific surface area [40]. Therefore, in this study, we used 2-methylimidazole as a ligand and silver nitrate as an ion provider to prepare a nano metal-organic framework named Ag-MOF, which was loaded with vancomycin to treat MRSA infection through a dual antibacterial mechanism.

In view of the short half-life of exogenous nanoparticles in the circulatory system, natural biofilm was used to camouflage nanoparticles in this study. Among natural biological carriers, platelets (PLT)—autologous blood cells—have good biocompatibility, with which synthetic carriers cannot compete [41]. Not only can platelet membrane-camouflaged nanoparticles significantly reduce the macrophage uptake and particle-induced complement activation, but also they still show platelet-like function after camouflaging, which can effectively solve the plasma protein absorption on nanomaterial surface [42, 43]. In addition, platelets are the first and most abundant cell type accumulated in the intravascular infection and have the ability to recognize inflammatory cells [44, 45]. The inflammatory endothelial cells express P-selectin and E-selectin; the platelet membrane can express VWF, ICAM-1, and P-selectin receptor GPIBα, thus enabling platelets to bind with the inflammatory endothelial cells [46]. *S. aureus* can bind to platelets through adhesins such as protein A, agglutinin A, agglutinin B, fibronectin A, and serine-rich surface proteins [47, 48]. Therefore, platelet membrane-camouflaged nanoparticles is expected to enhance the efficacy of existing drugs by protecting them from degradation; moreover, it can also increase the local drug concentration in infection site by providing targeted drug delivery and reinforce bactericidal effect. Nanoparticles targeting the infection site can deliver high concentration of antimicrobial drugs at the site of infection, while maintain a low total dose of drug administered, thus reducing the dose of antibiotics used and reducing toxicity to non-targeted organs.

**Fig. 1:**
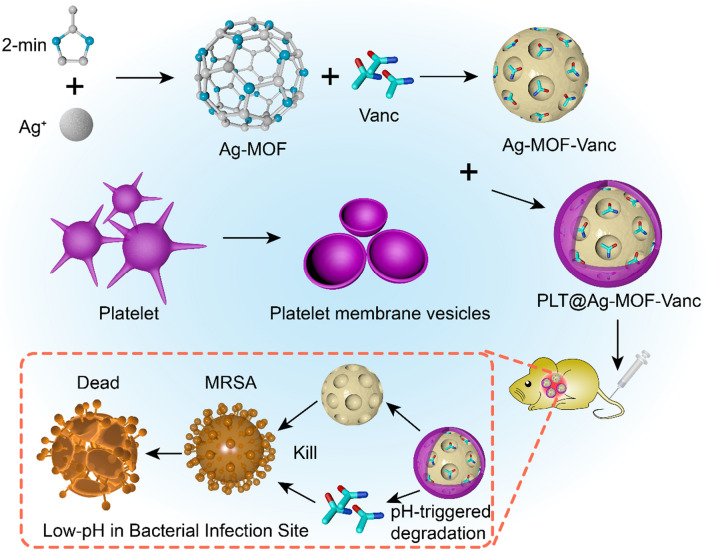
Schematic diagram of PLT@Ag-MOF-Vanc in the treatment for MRSA infection

In this study, nano-sized platelet vesicles were prepared to encapsulate Ag-MOF-Vanc, thereby forming the novel platelet membrane-camouflaged nanoparticles PLT@Ag-MOF-Vanc (Fig. 1). The PLT@Ag-MOF-Vanc synthesized in our study had a high drug loading rate, good biocompatibility, dual antibacterial mechanism, and targeting; it also has the ability to effectively improve the antibacterial activity of vancomycin, with no obvious toxicity.

## Results and discussion

### Preparation and characterization of PLT@Ag-MOF-Vanc

**Fig. 2:**
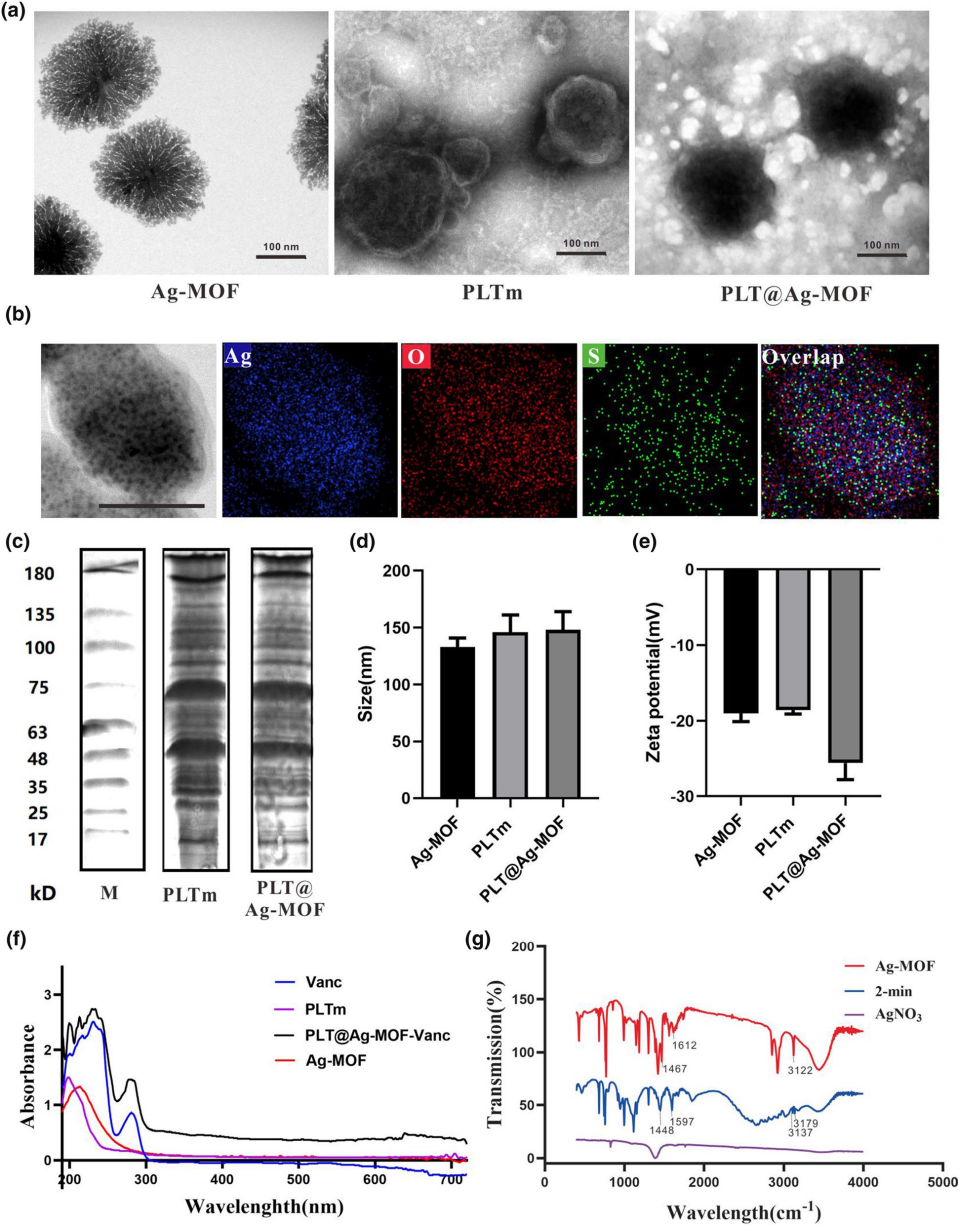
Characterization of PLT@Ag-MOF-Vanc. **a** TEM images of Ag-MOF, PLTm, and PLT@Ag-MOF. **b** TEM image and corresponding element mapping of PLT@Ag-MOF. Scale bar: 100 nm. **c** SDS-PAGE protein assessment. **d** Particle sizes and **e** zeta potential values of Ag-MOF, PLTm, and PLT@Ag-MOF. **f** UV-vis spectra of Vanc, Ag-MOF, PLTm, and PLT@Ag-MOF-Vanc. **g** FTIR spectra of Ag-MOF, 2-methylimidazole and AgNO_3_. Data are presented as means ± SD (n = 3)

As shown in Fig. 1, the preparation of PLT@Ag-MOF-Vanc mainly included the following three steps: (1) synthesis of Ag-MOF with 2-methylimidazole and AgNO_3_ in a high temperature reactor; (2) loading vancomycin to Ag-MOF to synthesize Ag-MOF-Vanc; (3) adding PLT membrane **(**PLTm) vesicles to encapsulate Ag-MOF-Vanc to form the final product PLT@Ag-MOF-Vanc. Transmission electron microscopy (TEM) (Fig. 2a) showed that the synthesized Ag-MOF particles had uniform size (130–150 nm) and shape of radiating corona. After the fusion with PLTm vesicles, Ag-MOF was observed to be encapsulated into PLTm vesicles. EDS element mapping for PLT@Ag- MOF (Fig. 2b) showed the colocalization of Ag atoms with elements (O and S) in PLTm, which could confirm the encapsulation of Ag-MOF by PLTm. The protein composition of PLTm vesicles and PLT@Ag-MOF-Vanc was detected by sodium dodecyl sulfate polyacrylamide gel electrophoresis(SDS-PAGE). The results showed that PLT@Ag-MOF-Vanc and PLTm vesicles had similar protein profiles, which also confirmed the successful transfer of PLTm proteins to PLT@Ag-MOF-Vanc (Fig. 2c). Dynamic light scattering (DLS) data showed that the particle size of Ag-MOF was about 133 nm. After the encapsulation by PLTm vesicles, the particle size of PLT@Ag-MOF-Vanc was about 148 nm and was close to the size of PLTm vesicles (146 nm, Fig. 2d), which was comparable with the TEM results. Zeta potential of Ag-MOF, PLTm, and PLT@Ag-MOF-Vanc were − 19.0 mV, − 18.6 mV, and − 25.6 mV, respectively (Fig. 2e).

UV-vis spectrum analysis showed that PLT@Ag-MOF-Vanc had three characteristic absorption peaks, located at 199 nm, 213 nm, and 281 nm, which were consistent with the characteristic absorption peaks of PLTm vesicles, Ag-MOF, and vancomycin, respectively (Fig. 2f). In the FTIR spectra of Ag-MOF (Fig. 2 g), the absorption peaks of imidazole ring in 2-methylimidazole all shifted to different degrees. C-H stretching vibration in the imidazole ring was shifted from 3137 cm^− 1^ to 3122 cm^− 1^, C-N stretching vibration in the imidazole ring was shifted from 1597 cm^− 1^ to 1612 cm^− 1^, and the stretching vibration of the imidazole ring was shifted from 1448 cm^− 1^ to 1467 cm^− 1^.This indicated that Ag ion in AgNO_3_ reacted with N in dimethyl imidazole to form Ag-N chemical bond, which changed the infrared spectrum absorption peak of imidazole ring in 2-methylimidazole. The N-H stretching vibration peak in the original 2-methylimidazole disappeared at 3179 cm^− 1^, indicating that Ag may have reacted with the N-H in the 2-methylimidazole.

### Drug loading and release of PLT@Ag-MOF-Vanc

**Fig. 3:**
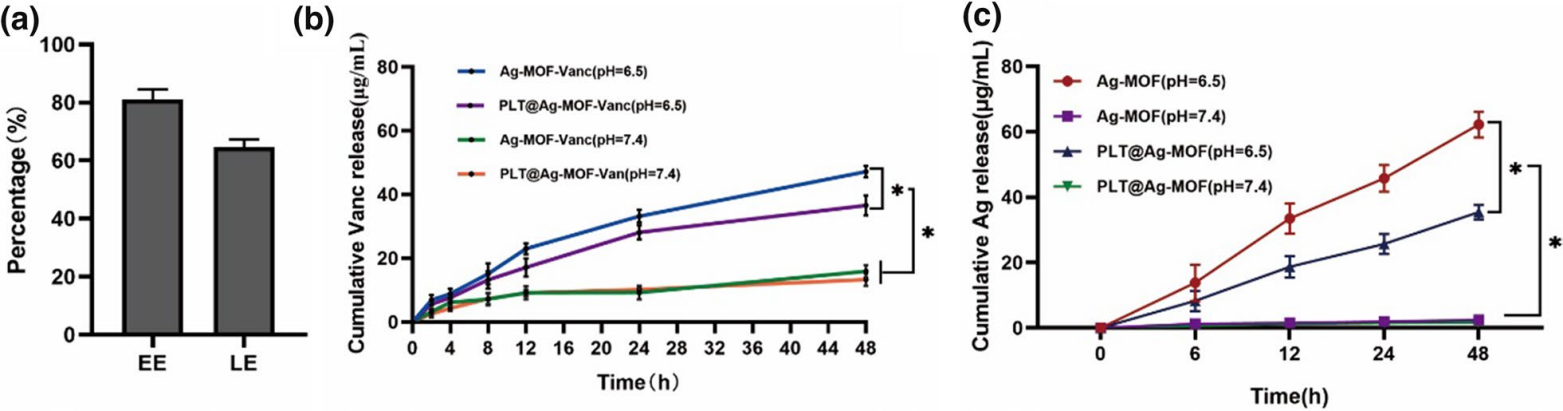
Drug loading and release of PLT@Ag-MOF-Vanc. **a** EE and LE of PLT@Ag-MOF-Vanc. **b** Cumulative release rates of vancomycin from Ag-MOF-Vanc or PLT@Ag-MOF-Vanc at different pH values (6.5 and 7.4). **c** Cumulative release rates of Ag^+^ from Ag-MOF-Vanc or PLT@Ag-MOF-Vanc at different pH values (6.5 and 7.4). Data are presented as means ± SD (n = 3). *p < 0.05

MOF is an ideal drug carrier due to its high porosity and specific surface area. In this study, vancomycin was loaded in Ag-MOF, and the encapsulation efficiency (EE) and loading efficiency (LE) of vancomycin were 81.0 and 64.7 %, respectively (Fig. 3a). The ideal drug carrier should efficiently load the drug but also reach specific sites to achieve responsive release. The pH values of the infected site and intracellular environment were lower than those of healthy tissue and extracellular environment, respectively [30, 49]. Therefore, pH-sensitive nanoparticles could have a better inhibitory effect on the infection of *S. aureus*. In order to verify the pH-responsive release of PLT@Ag-MOF-Vanc in infected microenvironment, pH 7.4 and pH 6.5 were used in this study to simulate neutral blood circulation environment and the acidic infection microenvironment, respectively. As shown in Fig. 3b, vancomycin was more easily released at pH 6.5 than at pH 7.4. It is beneficial for vancomycin in PLT@Ag-MOF-Vanc to be released at the infected site rather than in neutral circulation, indicating that PLT@Ag-MOF-Vanc can be used for drug delivery, especially at the infected site. Similar to vancomycin, the release rate of Ag^+^ in Ag-MOF increased with the decrease in pH value (Fig. 3c). In summary, Ag^+^ and vancomycin in PLT@Ag-MOF-Vanc can be released rapidly in the weak acidic environment of the infected area. In addition, the cumulative release rate of Ag^+^ and vancomycin in PLT@Ag-MOF-Vanc was lower than that in Ag-MOF-Vanc, indicating that PLTm inhibited the rapid release of the drug to some extent and played a role in continuous release. These results indicate that PLT@Ag-MOF-Vanc is an effective drug carrier.

### Biocompatibility of PLT@Ag-MOF-Vanc

**Fig. 4:**
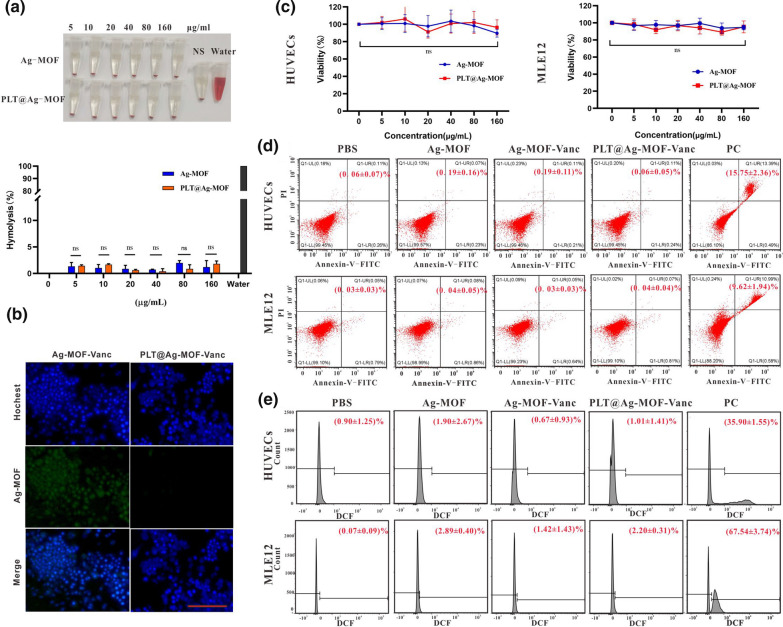
Biocompatibility of PLT@Ag-MOF-Vanc. **a** Images and hemolytic ratios of erythrocytes’ suspensions after treatment with different doses of Ag-MOF or PLT@Ag-MOF. **b** CLSM images of RAW264.7 cells upon culture with Ag-MOF-Vanc or PLT@Ag-MOF-Vanc for 24 h. Scale bar: 100 μm. **c **Cell viability of HUVECs and MLE12 cells treated with various concentrations of Ag-MOF and PLT@Ag-MOF based on CCK-8 test. **d** Apoptosis assessed by flow cytometry in HUVECs and MLE12 previously treated with Ag-MOF, Ag-MOF-Vanc, or PLT@Ag-MOF-Vanc for 48 h. **e** ROS level evaluation by flow cytometry in HUVECs and MLE12 treated with Ag-MOF, Ag-MOF-Vanc, or PLT@Ag-MOF-Vanc for 48 h. PC: Positive Control. Data are presented as means ± SD (n = 3). ns = no significance

To evaluate whether PLT@Ag-MOF-Vanc was endowed with good blood compatibility, hemolysis test was performed. Namely, 5 % erythrocytes were incubated with different concentrations of Ag-MOF and PLT@Ag-MOF (0, 5, 10, 20, 40, 80, and 160 µg/mL) for 2 h. As shown in Fig. 4a, Ag-MOF and PLT@Ag-MOF did not cause significant hemolysis during 2 h (less than 3 % for both). The results indicated that Ag-MOF itself had good blood compatibility, and could be maintained after PLTm encapsulation.

**Fig. 5:**
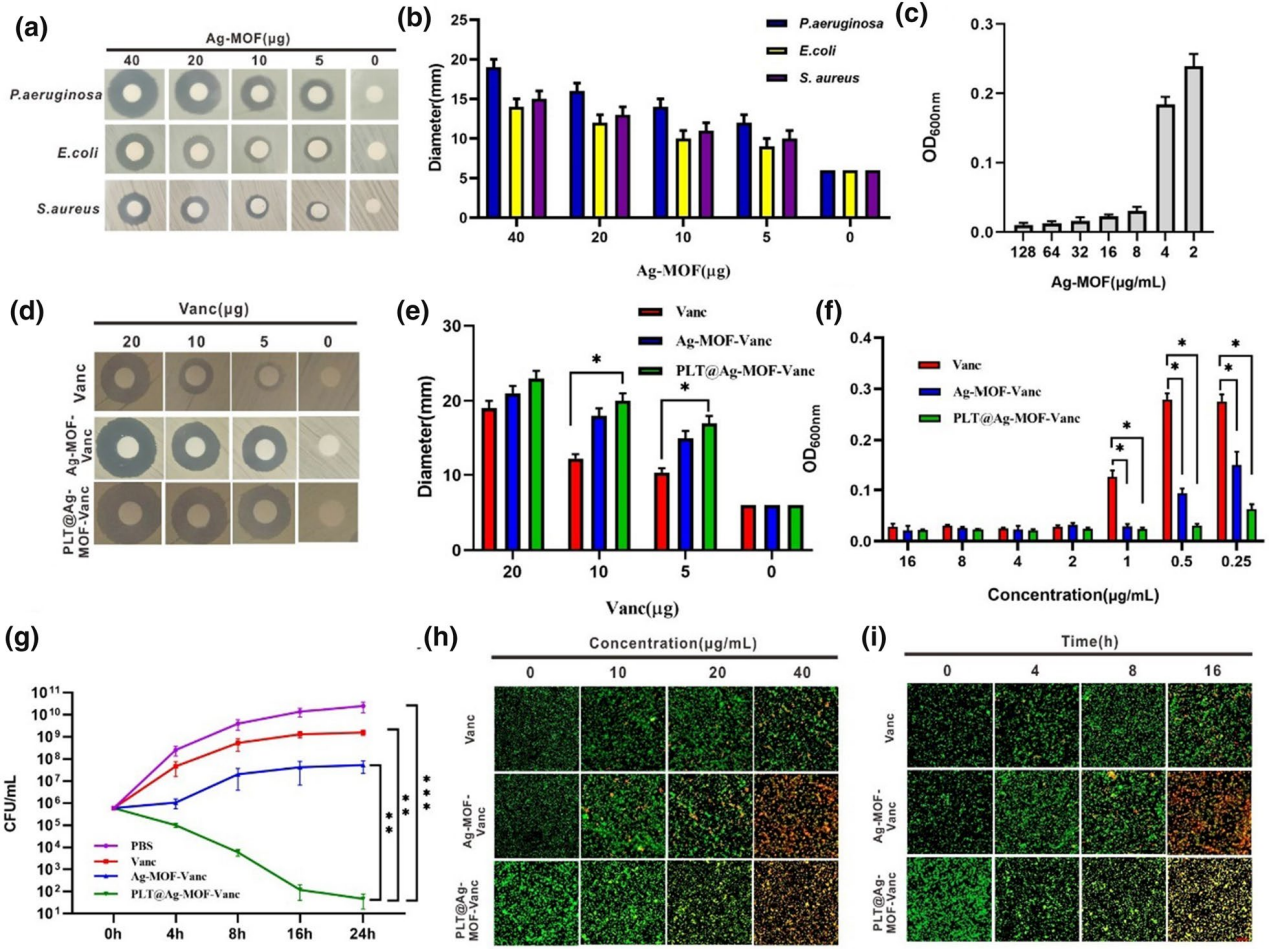
In vitro antibacterial effect of Ag-MOF-Vanc. **a** Inhibition zones and **b** corresponding inhibition zone diameters of Ag-MOF against different bacteria. **c **Concentration effects of Ag-MOF on the growth of MRSA. **d** Inhibition zones, **e** corresponding inhibition zone diameters and **f** concentration effects of Vanc, Ag-MOF-Vanc and PLT@Ag-MOF-Vanc against MRSA. **g** CFU of MRSA treated with 0.5 µg/mL of different drugs. **h** CLSM imaging of death/live staining after exposing MRSA to varying concentrations of Vanc, Ag-MOF-Vanc or PLT@Ag-MOF-Vanc. **i** CLSM imaging of death/live staining after MRSA exposure to Vanc, Ag-MOF-Vanc or PLT@Ag-MOF-Vanc with different incubation time. Scale bar: 20 μm. Data are presented as means ± SD (n = 3).* p < 0.05, **p < 0.01, ***p < 0.001

To demonstrate the immune escape ability of PLTm-camouflaged PLT@Ag-MOF-Vanc, the phagocytosis of RAW264.7 macrophages was evaluated by confocal laser scanning microscope (CLSM). The green fluorescence of Ag-MOF was used for cell imaging. As shown in Figs. 4b and 5 h after injection of Ag-MOF-Vanc, a large number of Ag-MOF was engulfed by RAW264.7 cells. Meanwhile, under the same conditions, the green fluorescence in RAW264.7 cells treated with PLT@Ag-MOF-Vanc was significantly reduced; these data indicated that after being encapsulated by PLTm vesicles, the immunogenicity of PLT@Ag-MOF-Vanc decreased and was not recognized as a non-self-component by macrophages, so the phagocytosis was effectively inhibited. These above characteristics endowed PLT@Ag-MOF-Vanc with prolonged circulatory half-life by reducing recognition and clearance by phagocytes from the reticuloendothelial system in vivo.

To further evaluate the cytotoxicity of the material, HUVECs and MLE12 cells were treated with different concentrations (0, 5, 10, 20, 40, 80, and 160 μg/mL) of Ag-MOF and PLT@Ag-MOF, and the cell vitality was detected by CCK-8. As shown in Fig. 4c, after 48 h of mixed culture with the added material, cell vitality did not significantly decrease; therefore, Ag-MOF and PLT@Ag-MOF have no obvious cytotoxicity. In this study, the effects of the materials on cell apoptosis and reactive oxygen species (ROS) production were further detected by flow cytometry. Compared with the phosphate buffer solution(PBS) group, the apoptosis rate and ROS production were not increased in Ag-MOF, Ag-MOF-Vanc, and PLT@Ag-MOF-Vanc groups (Fig. 4d and e). Apoptosis is a mechanism of cell death, and ROS is a key molecule in cell apoptosis and autophagy [50]. These results indicate that the synthesized material in this study does not lead to cell death by promoting apoptosis.

### In vitro antibacterial effect of Ag-MOF-Vanc

In this study, the antibacterial effect of the newly synthesized Ag-MOF on three common clinical strains (*Escherichia coli* ATCC25922, *Pseudomonas aeruginosa* ATCC27853, and *S. aureus* ATCC25923) was investigated by disc method. The results of antibacterial zone showed that Ag-MOF had a good inhibitory effect on the growth of the three strains (Fig. 5a and b). For MRSA (ATCC25923), the minimum inhibitory concentration (MIC) of Ag-MOF was 8 µg/mL (Fig. 5c). The antibacterial effects of free vancomycin, Ag-MOF-Vanc and PLT@Ag-MOF-Vanc on MRSA were compared; the results showed that the antibacterial zones of Ag-MOF-Vanc and PLT@ Ag-MOF-Vanc were larger than that of free vancomycin at different concentrations (Fig. 5d and e). The MIC of free vancomycin was 2 µg/mL; in contrast, the MIC of Ag-MOF-Vanc was 1 µg/mL, and the MIC of PLT@ Ag-MOF-Vanc was 0.5 µg/mL (Fig. 5f), which was much lower than that of free vancomycin. Then, we co-incubated MRSA with 0.5 µg/mL free vancomycin, Ag-MOF-vanc or PLT@Ag-MOF-Vanc, and colony forming unit (CFU) of bacteria was detected at 0, 4, 8, 16 and 24 h, respectively. The results showed that although the growth of MRSA in free vancomycin and Ag-MOF-vanc groups were lower than that in PBS group, the bacteriostatic effect was not significant, while the bacterial growth in PLT@Ag-MOF-Vanc group was significantly inhibited (Fig. 5g). The above results indicated that Ag-MOF could enhance the antibacterial effect of vancomycin against MRSA and reduce the dosage of vancomycin.

MRSA was exposed to different concentrations of drugs or for different periods of time, then stained with the Live/Dead backlight bacterial viability kit, and the images were measured with CLSM. When MRSA was exposed to different concentrations (0, 10, 20, 40 µg/mL) of Vanc, Ag-MOF-Vanc and PLT@ Ag-MOF-Vanc for 1 h, the number of dead bacteria increased in a dose-dependent manner, suggesting that the antibacterial activity of PLT@Ag-MOF-Vanc was concentration-dependent (Fig. 5h). MRSA was treated with 1 µg/ mL vancomycin, Ag-MOF-Vanc and PLT@ Ag-MOF-Vanc. The permeability of the bacteria increased with prolonged time, suggesting the time dependence of PLT@ Ag-MOF-Vanc (Fig. 5i).

### Antibacterial mechanism of PLT@Ag-MOF-Vanc

**Fig. 6:**
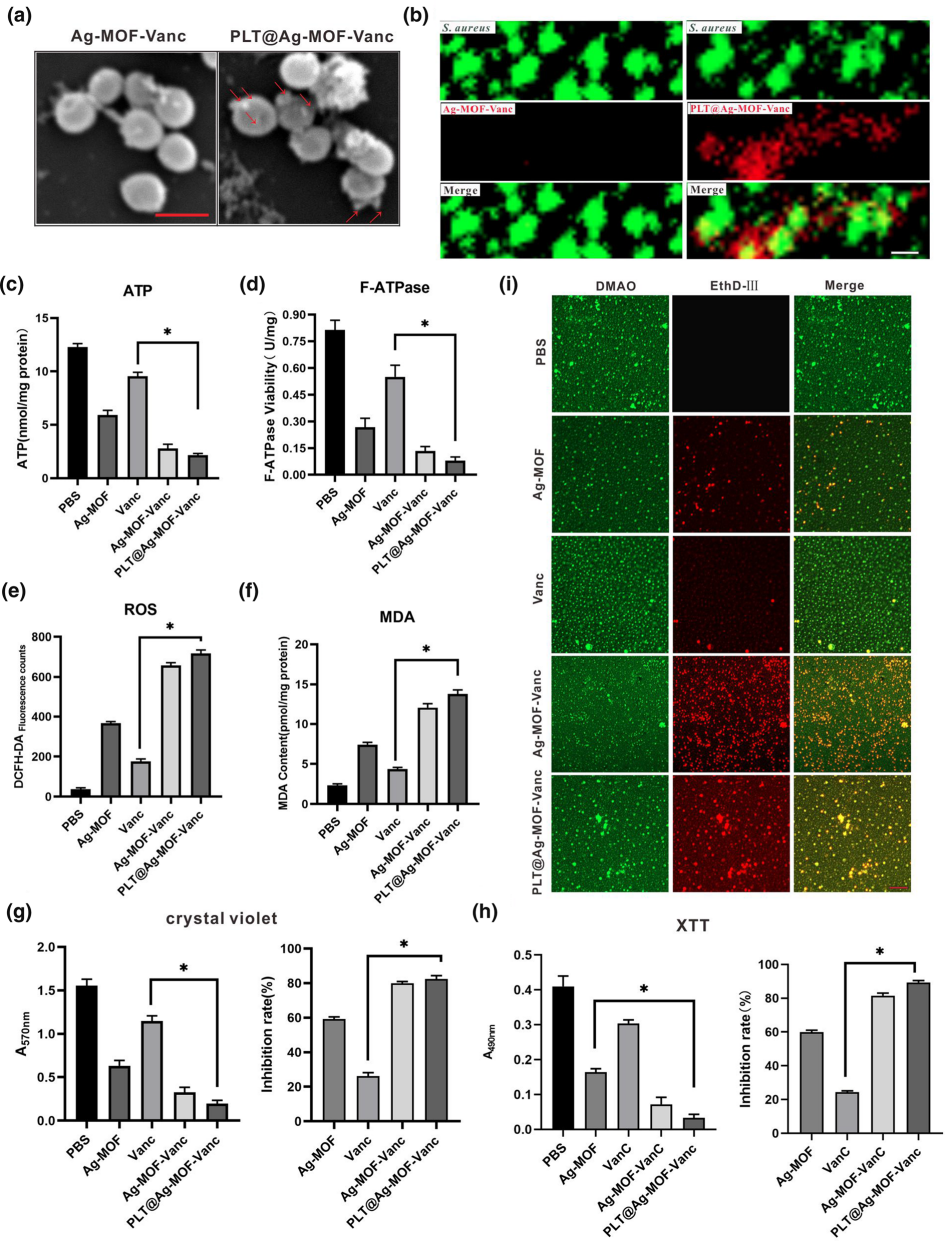
Antibacterial mechanism of PLT@Ag-MOF-Vanc. **a** SEM images of MRSA incubated with Ag-MOF-Vanc or PLT@ Ag-MOF-Vanc. Scale bar: 1 μm **b **CLSM images of MRSA incubated with Cy5-labeled Ag-MOF-Vanc and Cy5-labeled PLT@Ag-MOF-Vanc. Scale bar: 2 μm. **c** Intracellular ATP level, **d** F-type ATPase activity, (3) DCFH-DA fluorescence counts, and **e** MDA contents of MRSA treated with Ag-MOF, vancomycin, Ag-MOF-Vanc, or PLT@ Ag-MOF-Vanc. **g** Crystal violet staining, **h** XTT dyeing, and **i** CLSM imagaes of MRSA biofilm. Scale bar: 20 μm. Data are presented as means ± SD (n = 3). *p < 0.05

A series of studies were conducted to explore the antibacterial mechanism of PLT@Ag-MOF-Vanc. The first step for PLT@Ag-MOF-Vanc to exert its antibacterial effect is to target MRSA with the assistance of PLTm. In order to clarify the interaction between PLT@Ag-MOF-Vanc and MRSA, Ag-MOF-Vanc and PLT@Ag-MOF-Vanc at a certain concentration were co-incubated with MRSA for 3 h and observed by Scanning electron microscopy (SEM). Figure 6a shows that the surface of MRSA was relatively smooth when exposed to Ag-MOF-Vanc, while a large number of nanoparticles were attached to the surface after the exposure to PLT@Ag-MOF-Vanc. This indicated that PLTm promoted the binding of PLT@Ag-MOF-Vanc to MRSA and had a certain targeting effect. In order to observe the binding of the nanomaterials to bacteria more clearly, we co-incubated Cy5-labeled Ag-MOF-Vanc or Cy5-labeled PLT@Ag-MOF-Vanc with MRSA which were stained with DMAO, and observed with CLSM. Figure 6b showed the binding of PLT@Ag-MOF-Vanc to cultured *S. aureus* in vitro, in contrast to Ag-MOF-Vanc without bacterial binding.

To evaluate the effect of PLT@Ag-MOF-Vanc on bacterial metabolism, intracellular ATP levels were measured. The results showed that PLT@Ag-MOF-Vanc led to a significant decrease in ATP level, which was significantly greater than that caused by Ag-MOF and vancomycin (Fig. 6c). The decrease in ATP levels may be attributed to the inactivation of F-type ATP synthase (F-ATPase) (Fig. 6d). The functions of F-ATPase include catalyzing the synthesis of ATP in the last step of oxidative phosphorylation, working in reverse as an ATPase to produce the transmembrane proton electrochemical gradient required for molecular transport [51]. PLT@Ag-MOF-Vanc could significantly decrease the activity of F-ATPase. F-ATPase is widely expressed in mammalian cells, but as shown in Fig. 4c, Ag-MOF did not affect the cell viability. It can be indicated that Ag-MOF showed selective toxicity to bacteria but not to mammalian cells. This selectivity may be due to the fact that Ag-MOF is difficult to affect the respiratory chain in mammalian mitochondria, which need to overcome several barriers including the escape from lysosomes, targeting mitochondria, and entry into of mitochondria through the membrane [52, 53].

The death of bacteria exposed to nanoparticles can be attributed to the disruption of energy production caused by the decoupling of oxidized phosphate in the cellular respiratory chain, the interference in membrane permeability, and the loss of enzyme activity involved in key metabolic pathways; among them, the excessive ROS production by cells is the most effective component for triggering bacterial cell death [54]. Therefore, it is very important to study the effect of nanoparticles on the formation of ROS in bacterial cells. We used 2,7-dichlorodihydrofluorescein diacetate (DCFH-DA) method to quantitatively detect ROS. Figure 6e shows the ROS production level of MRSA after the treatment with Ag-MOF, free vancomycin, Ag-MOF-Vanc, and PLT@Ag-MOF-Vanc. Compared with the control group and the free vancomycin group, Ag-MOF treatment significantly increased ROS levels. The variation was more obvious in the bacteria treated with Ag-MOF-Vanc and PLT@ Ag-MOF-Vanc. High ROS levels were observed in PLT@Ag-MOF-Vanc-treated bacteria, indicating that PLT@Ag-MOF-Vanc effectively bound to the bacterial surface, thus releasing a high proportion of silver ions in the target cells.

One of the main consequences of intracellular ROS accumulation is the damage to the membrane integrity caused by the gradual establishment of oxidative stress. In addition, nanoparticles can also cause physical damage to the cell membrane. Therefore, we continued to use malondialdehyde (MDA) method to detect cell lipid peroxidation to determine the degree of membrane damage. There were significant differences in MDA content among bacteria in different treatment groups (Fig. 6f). Compared with free vancomycin, the content of MDA in PLT@Ag-MOF-Vanc-treated cells increased significantly. These results suggested that the interaction between Ag-MOF and bacterial surface increased the degree of bacterial damage.

One of the reasons for drug resistance and poor therapeutic effect of antibiotics is the generation of biofilms. Because of their high permeability, nanoparticles can penetrate thick biofilms. We speculated that Ag-MOF-Vanc may have a good inhibitory and scavenging effect on *S. aureus* biofilm. During the early- and mid-stages during biofilm formation, two assays, crystal violet staining and the 2,3-bis (2-methoxy-4-nitro-5-sulfophenyl)-2 H-tetrazolium-5-carbox-anilide (XTT) assay, are regarded as crucial experimental tools. The results of crystal violet staining and XTT staining after treating the MRSA biofilm with different drugs showed that PLT@Ag-MOF-Vanc effectively destroyed the biofilm formed by MRSA, and the effect was obviously better than that of Ag-MOF and vancomycin alone (Fig. 6g and h). Bacterial biofilm formation was also confirmed by using LIVE/DEAD backlight bacterial viability staining and observed by CLSM. As shown in Fig. 6i, after 24 h of treatment with PLT@Ag-MOF-Vanc, there were maximum fluorescence intensities of red fluorescence, which reflected that the PLT@Ag-MOF-Vanc killed biofilms of MRSA. The results obtained by CLSM were identical to those obtained by crystal violet and XTT analysis.

In conclusion, PLT@Ag-MOF-Vanc could kill MRSA through a comprehensive physical and chemical mechanism, including targeting MRSA via PLTm; interfering with the intracellular metabolism of bacteria; catalytic production of ROS; damage to cell membrane integrity; and inhibiting the formation of biofilm.

### Distribution of intravenously injected PLT@ Ag-MOF-Vanc

**Fig. 7:**
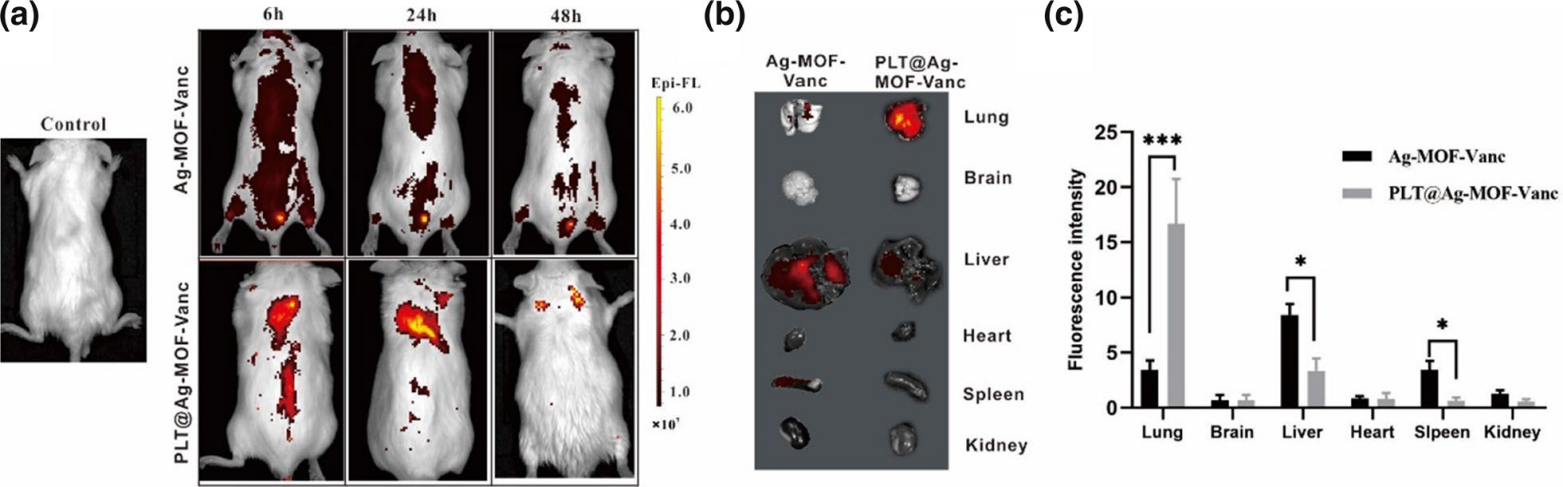
In vivo targeting potential of PLT@Ag-MOF-Vanc. **a** In vivo fluorescence images of MRSA pneumonia mice at 6, 24, and 48 h upon intravenous treatment with Cy5-labeled Ag-MOF-Vanc and Cy5-labeled PLT@Ag-MOF-Vanc. **b** Ex vivo bioluminescence images of visceral organs at 48 h after treatment with Cy5-labeled Ag-MOF-Vanc and Cy5-labeled PLT@Ag-MOF-Vanc. **c** Semiquantitative assessment of fluorescence signals of visceral organs. Control = mouse MRSA pneumonia model without any injection. Data are mean ± SD (n = 3). *p < 0.05, ***p < 0.001

To demonstrate that PLT@Ag-MOF-Vanc can target the MRSA-infected sites in vivo, biodistribution in MRSA pneumonia model mice was evaluated by Small Animal In Vivo Imaging at 6, 24, and 48 h after Ag-MOF-Vanc and PLT@Ag-MOF-Vanc tail vein injection. As shown in Fig. 7a, there is a significant difference in the distribution of Ag-MOF-Vanc and PLT@Ag-MOF-Vanc. PLT@Ag-MOF-Vanc was significantly accumulated in lungs within 48 h of administration and seldom accumulated in brain, heart, liver, spleen and kidney, while Ag-MOF-Vanc rarely aggregated at the infected site within 48 h after injection. In the Ag-MOF-Vanc group, there were obvious signals in the bladder, which might attribute to the accumulation of Ag-MOF-Vanc in the bladder after metabolism by the kidney and then excreted in the urine. 48 h after injection, the mice were killed by cervical dislocation, and fluorescence imaging of the lung, brain, liver, heart, spleen and kidney was performed ex vivo (Fig. 7b and c). There was a small amount of Ag-MOF-Vanc aggregation in the lung, liver, and spleen; the accumulation of PLT@Ag-MOF-Vanc in the lung was much higher than that of Ag-MOF-Vanc. The above results indicated that PLT@Ag-MOF-Vanc has a good targeting effect on the MRSA-infected sites in vivo.

### Anti-infection effect of PLT@Ag-MOF-Vanc in vivo

**Fig. 8:**
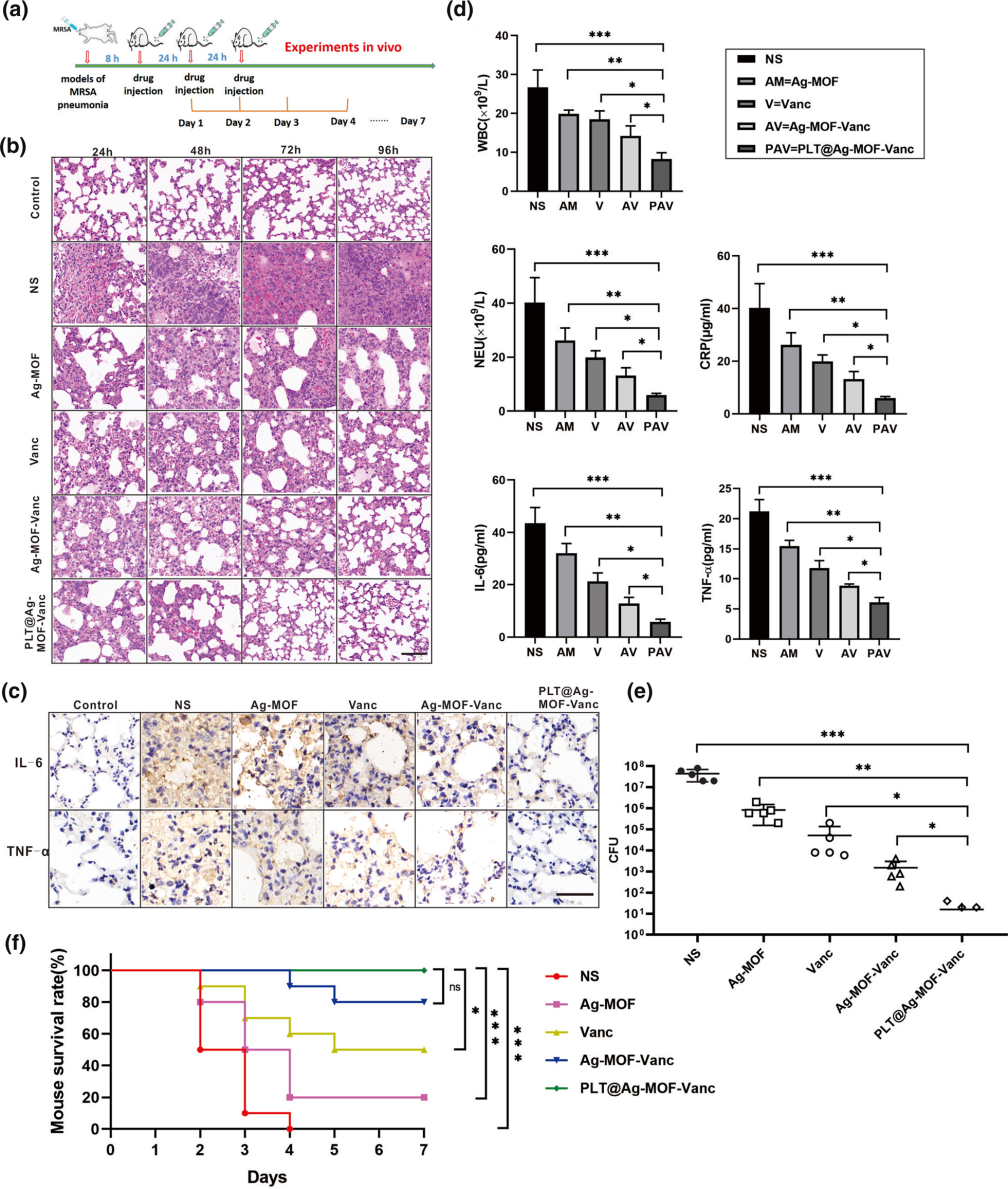
Anti-infection effect of PLT@Ag-MOF-Vanc in vivo. **a** Schematic diagram of in vivo anti-infection experimental design. **b** HE staining images of lung tissues. **c** Immunohistochemical staining of IL-6 and TNF-α in lung tissues. **d** Inflammation-associated cell counts and cytokine levels in the blood. Data are presented as means ± SD (n = 5). **e** Bacterial count in alveolar lavage fluid (n = 5). **f** Survival rates in the mouse MRSA pneumonia model after tail-vein injection of different drugs (n = 10). Control = normal healthy mice. Scale bar: 100 μm. *p  < 0.05, **p < 0.01, ***p < 0.001

The present study further evaluated the anti-infection effect of PLT@Ag-MOF-Vanc in vivo in the MRSA pneumonia model of Kunming mice. The infected mice were divided into the following five groups: normal saline group, Ag-MOF group, vancomycin group, Ag-MOF-Vanc group, and PLT@Ag-MOF-Vanc group. We carried out the anti-infection experiments in vivo according to the Fig. 8a. After establishing the model, the corresponding drugs were injected daily. According to the vancomycin concentration, the dosage was 2 mg/kg every day for vancomycin group, Ag-MOF-Vanc group, and PLT@Ag-MOF-Vanc group. The dosage of Ag-MOF group was the same as the concentration of Ag-MOF contained in Ag-MOF-Vanc and PLT@Ag-MOF-Vanc groups. Every day one mouse was taken from each group for Hematoxylin-eosin(HE) staining so as to observe the alveolar structure and integrity of ciliated endothelial cells, inflammation, necrosis, and infiltration by inflammatory cells (macrophages) in the alveoli. The results showed significantly better improvement rate of the lung condition in PLT@Ag-MOF-Vanc group compared with other groups, and the alveoli recovered from the third day of the treatment, with no obvious inflammatory cell infiltration (Fig. 8b). Four days after the treatment, the levels of inflammatory cytokines interleukin-6 (IL-6) and tumor necrosis factor-α (TNF-α) in the lung tissue of mice were examined by immunohistochemical staining; the results showed that the expression levels of IL-6 and TNF-α in the normal saline group, Ag-MOF group, vancomycin group, and Ag-MOF-Vanc group were still significantly higher compared with the normal control mice, while those in PLT@Ag-MOF-Vanc group almost returned to normal control level (Fig. 8c). In addition, blood was taken for hematological tests; the results showed that the white blood cell (WBC), neutrophil (NEU) count and inflammatory marker C-reactive protein (CRP) level were significantly reduced in the PLT@Ag-MOF-Vanc group (Fig. 8d). The levels of IL-6 and TNF-α in blood were examined by enzyme-linked immuno sorbent assay (ELISA); it was shown that the level of inflammatory cytokines significantly decreased in the PLT@Ag-MOF-Vanc group (Fig. 8d). The bacterial count in alveolar lavage fluid of different treatment groups also showed that the number of residual bacteria was the lowest after the treatment with PLT@Ag-MOF-Vanc (Fig. 8e).

We reprepared a group of mice infected with MRSA pneumonia and randomized them to receive corresponding treatment to observe the survival rate of each group. MRSA killed all mice treated with saline, and different numbers of mice died within 7 days in the Ag-MOF group, vancomycin group and Ag-MOF-Vanc group, but the survival rate of mice in the PLT@Ag-MOF-Vanc group was 100 % (Fig. 8f). In the PLT@Ag-MOF-Vanc group, the concentration of vancomycin was 2 mg/kg, while the survival rate in the free vancomycin group (2 mg/kg) was only 50 %, indicating that under the same dose of vancomycin, the therapeutic effect of PLT@Ag-MOF-Vanc was better than that of free vancomycin. According to the literature [39], in the treatment of mouse pneumonia model, vancomycin should reach at least 15 mg/kg, and the survival rate can reach 100 %, indicating that PLT@Ag-MOF-Vanc involved in this study can effectively reduce the dose of vancomycin.

All the above results show that PLT@Ag-MOF-Vanc has a good anti-infective effect in mouse MRSA pneumonia model, which is significantly superior to free vancomycin and Ag-MOF alone or uncoated Ag-MOF-Vanc, indicating that Ag-MOF and vancomycin have a synergistic anti-infective effect. Meanwhile, after encapsulation with PLTm, PLT@Ag-MOF-Vanc could be targeted and transported to the MRSA-infected site, thus further strengthening the anti-infective effect of PLT@Ag-MOF-Vanc.

### In vivo toxicity assessment of PLT@Ag-MOF-Vanc

**Fig. 9:**
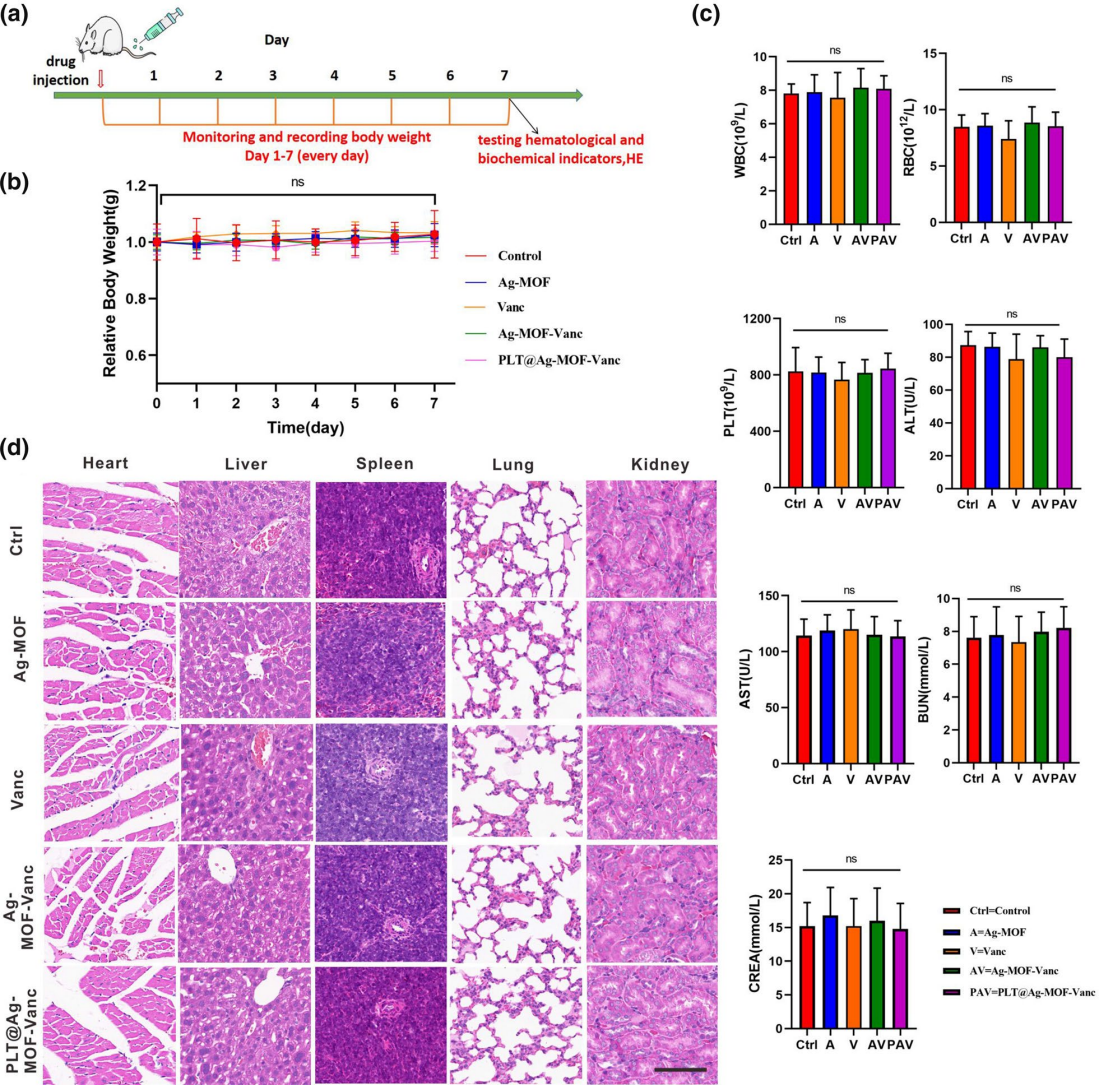
In vivo toxicity assessment of PLT@Ag-MOF-Vanc. **a** Schematic diagram of in vivo toxicity experimental design. **b** Mice weight changes after injecting with normal saline, Ag-MOF, vancomycin, Ag-MOF-Vanc, and PLT@ Ag-MOF-Vanc during 1 week. **c** CBC and serum indexes in mice at 1 week after intravenous injection of corresponding drugs. **d** Histological images of heart, liver, spleen, lung, and kidney samples from mice at 1 week after intravenous injection of corresponding drugs. Scale bar: 100 μm. Control: mice treated with normal saline. Data are indicated as mean ± SD (n = 5). Ns = no significance

In order to evaluate the potential toxicity of PLT@Ag-MOF-Vanc, healthy Kunming rats were randomized into five groups and injected with 100 µL of normal saline, Ag-MOF, vancomycin, Ag-MOF-Vanc and PLT@Ag-MOF-Vanc. According to the vancomycin concentration, the dosage was 2 mg/kg for vancomycin group, Ag-MOF-Vanc group, and PLT@Ag-MOF-Vanc group. The dosage of Ag-MOF group was the same as the concentration of Ag-MOF contained in Ag-MOF-Vanc and PLT@Ag-MOF-Vanc groups. As shown in the Fig. 9a, we designed the toxicity experiments in vivo. We recorded the changes in mouse body weights every day for 1 week after tail vein drug injection, and found no significant difference in body weight among the five groups (Fig. 9b). Hematological indicators of normal mice were measured 1 week after the tail vein drug injection. We measured complete blood counts (red blood cell (RBC), WBC, platelet (PLT)), liver function indicators (alanine transaminase (ALT), aspartate aminotransferase (AST)), and renal function indicators (blood urea nitrogen (BUN), and creatinine (CREA)). There was no significant difference in any of these indicators among different groups (Fig. 9c), indicating that PLT@Ag-MOF-Vanc had no significant effect on the production of red blood cells, white blood cells, and platelets in blood, and it had no obvious hepatorenal toxicity.

To further clarify the in vivo toxicity of PLT@Ag-MOF-Vanc, we used HE staining to evaluate the pathological changes in major organs (heart, liver, spleen, lung, and kidney) and we didn’t observe any arrested necrosis, fibrosis, histological abnormalities, and inflammatory lesions among the five groups (Fig. 9d). The results demonstrated that PLT@Ag-MOF-Vanc did not cause significant damage to the heart, liver, spleen, lung, and kidney; it had low toxicity in vivo and good biocompatibility.

## Conclusions

In this study, we designed and synthesized a PLTm-camouflaged nano-drug delivery system (PLT@Ag-MOF-Vanc). The drug release and silver ion release of PLT@Ag-MOF-Vanc were shown to be pH-responsive, which can prevent the delivery system from prereleasing drugs in the circulatory system. The platelet membrane on PLT@Ag-MOF-Vanc effectively covers the internal nano-core, reduces the recognition and clearance of reticuloendothelial system, and has good biocompatibility. Ag-MOF-Vanc showed good antibacterial activity against common clinical strains in vitro, significantly better than free vancomycin. In the MRSA pneumonia model, PLT@Ag-MOF-Vanc targeted the MRSA-infected sites. Combining the antibacterial ability of Ag-MOF with vancomycin, the growth of MRSA was inhibited and the effect was better than when using vancomycin alone; in that way, when the same dosage of vancomycin was used, the therapeutic effect of PLT@Ag-MOF-Vanc was significantly better than that of free vancomycin, and no obvious toxicity was observed. PLT@Ag-MOF-Vanc is a novel and effective targeted drug delivery system that can be used for safe and effective anti-infection therapy.

## Materials and methods

### Materials

Silver nitrate and 2-methylimidazole were purchased from Aladdin (China). Cy5 and Hoechst 33,342 were provided by Yeasen Biotechnology (China) Co., Ltd. Dialysis membrane (2 kD) was purchased from SolarBio (China). Annexin V-FITC/PI Apoptosis assay Kit and ROS assay Kit were provided by Beyotime Biotechnology (China). Cell Counting Kit-8 (CCK-8) was manufactured by Dojindo Laboratories, Japan. Primary antibodies against TNF-α and IL-6 monoclonal antibodies were purchased from Proteintech (China). Viability/Cytotoxicity Assay for Bacteria Live and Dead Cells was purchased from US Everbright Inc. (USA).

### Cells and mice

HUVECs, MLE12 and RAW264.7 cells were bought from the Advanced Research Center, Central South University; they were cultured in RPMI-1640 medium supplemented with 10 % fetal bovine serum. The cells were kept at 37 °C in 5 % CO_2_ at atmospheric pressure. Kunming mice (male, 8 weeks old) were bought from Hunan SJA Laboratory Animal Co., Ltd.

### Synthesis of Ag-MOF

First, 1 g 2-methylimidazole was dissolved in 10 mL ddH_2_O; 57 mg silver nitrate was dissolved in 2 mL ddH_2_O; then, the silver nitrate solution was dropped into the 2-methylimidazole aqueous solution. The solution was placed in a polyethylene reaction pot at 120 °C for 15 min, washed 2–3 times using ddH_2_O, and centrifuged at 14,000 rpm for 10 min.

### Synthesis of Ag-MOF-Vanc

We dissolved 0.5 mg Ag-MOF in 1 mL ddH_2_O; 5.2 µg vancomycin was added, stirred overnight at room temperature by magnetic force, and Ag-MOF-Vanc was obtained by centrifugation.

### Preparation of PLTm vesicles

The whole blood of male Kunming mice was used and placed in tubes containing heparin. Platelets were isolated from whole blood by centrifugation and washing. PLTm were extracted by repeated freeze–thaw extraction, suspended in PBS, and then subjected to ultrasonic treatment (2 min, 42 kHz, 100 W) to produce PLTm vesicles.

### Construction of PLT@Ag-MOF-Vanc

PLTm vesicles were fused with equal volume of Ag-MOF-Vanc by ultrasound (5 min, 42 kHz, 100 W). The sample was filtered 20 times using a porous syringe filter with a membrane pore diameter of 200 nm and centrifuged (2500 rpm, 10 min) to remove the excess PLTm to separate PLT@Ag-MOF-Vanc.

### Characterization

Ag-MOF, PLTm and PLT@Ag-MOF were observed through TEM with Tecnai G2 Spirit TEM (FEI, USA) to confirm that the PLTm was wrapped on the surface of Ag-MOFs. Elemental mapping analysis was performed by FEI Talos F200x with an integrated Super-X EDS system (FEI, USA). Zetasizer Nano ZS (Malvern Nano Series, Malvern, UK) was used to evaluate the particle size distribution and zeta potential. The PLTm proteins were identified by SDS-PAGE. The absorption spectra of Ag-MOF-Vanc were obtained using UV-Vis (Scandrop, Analytik Jena, Germany). The molecular functional groups of Ag-MOF-Vanc were studied by Fourier-transform infrared spectroscopy (FTIR).

### Determination of EE and LE

We dissolved 0.5 mg Ag-MOF in 1 mL ddH_2_O, and 5.2 µg vancomycin was added and stirred overnight by magnetic stirring at room temperature. Ag-MOF-Vanc was obtained by centrifugation. The maximum absorbance of vancomycin was determined by UV-vis spectrophotometry. The standard concentration gradient was set and the standard curve of concentration and absorbance was established; then, we calculated the uncombined vancomycin in the supernatant. The calculation formulas for LE and EE were as follows:

$$\text{EE}=(\text{quality}\;\text{of}\;\text{drugs}\;\text{contained}\;\text{on}\;\text{nano-carrier}/\text{total}\;\text{amount}\;\text{of}\;\text{drugs}\;\text{used})\;\times\;100\:\%,$$

$$\text{LE}=(\text{mass}\;\text{of}\;\text{drug}\;\text{contained}\;\text{on}\;\text{nano-carrier}/\text{mass of nano-carrier})\;\times\;100\:\%.$$

### Release of vancomycin and silver ion

In vitro drug release experiments were carried out under pH 7.4 and pH 6.5 to observe whether PLT@Ag-MOF-Vanc could release vancomycin and silver ion more easily under acidic environment. Next, 2 mL PLT@Ag-MOF-Vanc was put into a 2kD dialysis bag and immersed in 20 mL PBS solution at pH 7.4 and pH 6.5, seperately. The absorbance of vancomycin in dialysate was determined at 280 nm. Cumulative release of vancomycin was based on the standard curve. The concentration of Ag^+^ in dialysate was determined by a direct-reading inductively coupled plasma emission spectrometer (Spectro Blue, Spectro, Germany).

### Biocompatibility of PLT@Ag-MOF-Vanc

The biocompatibility of PLT@Ag-MOF-Vanc was evaluated by hemolysis rate and macrophage phagocytosis test. PLT@Ag-MOF-Vanc specimens of different concentrations (5–160 µg/mL) were mixed with 5 % Kunming mice erythrocyte suspension, incubated at 37 °C for 2 h, and centrifuged at 3500 rpm for 5 min. The absorbance was obtained at 545 nm using ultra-pure water and normal saline as positive and negative controls, respectively. The evaluation formula of hemolysis rate was as follows: hemolysis rate (%) = (absorbance of experimental sample − absorbance of negative control)/(absorbance of positive control − absorbance of negative control) × 100. To determine the immune escape ability of PLT@Ag-MOF-Vanc, RAW264.7 cells were inoculated in a 6-well plate (about 3 × 10^5^ cells/well) and incubated for 24 h. PLT@Ag-MOF-Vanc and Ag-MOF-Vanc were added and incubated for 24 h and then stained with Hoechst 33,342. The phagocytosis and fluorescence signals of macrophages against PLT@Ag-MOF-Vanc were obtained under CLSM (Zeiss LSM 800, Germany).

The cytotoxicity of PLT@Ag-MOF-Vanc was evaluated using HUVECs and MLE12 cells. The cells were inoculated in 96-well plate (2 × 10^3^ cells/well) and incubated for 24 h. The concentrations of Ag-MOF-Vanc and PLT@Ag-MOF-Vanc were calculated with the concentration of vancomycin as 20.0 µg/mL. The dosage of Ag-MOF group was the same as the concentration of Ag-MOF contained in Ag-MOF-Vanc and PLT@Ag-MOF-Vanc groups. After incubation for 48 h, CCK-8 solution (10 µL) was added to each well for 3 h, and the absorbance was measured at 450 nm.

Cell apoptosis was detected by Annexin V-FITC/PI apoptosis detection kit. In brief, the cells were inoculated at 10^6^ cells/bottle in T25 culture containers and incubated for 24 h until the cells adhered to the wall. Ag-MOF, Ag-MOF-Vanc and PLT@Ag-MOF-Vanc were added for further incubation for 48 h. Apoptosis was detected by flow cytometry as per standard procedures of the Annexin V-FITC/PI Apoptosis Detection Kit.

The production of ROS was detected. The cells were inoculated at 10^6^ cells/bottle in T25 culture containers and incubated for 24 h until the cells adhered to the wall. Ag-MOF, Ag-MOF-Vanc and PLT@Ag-MOF-Vanc were added for further incubation for 48 h. After additional incubation for 48 h, the intracellular ROS were analyzed by flow cytometry in accordance with the standard operation of the ROS detection kit.

### In vitro antibacterial experiment

#### Disc diffusion method

After single bacterial colony was obtained, the concentration of the bacterial solution was adjusted to 0.5 MCF with normal saline, and the bacterial solution was evenly spread on Mueller-Hinton (MH) medium. After placing a paper on the staining medium, different contents of the Ag-MOF, vancomycin, or Ag-MOF-Vanc were injected into the paper. After incubation for 1 day, the inhibition zone was observed.

#### Dilution method

First, we took one to two MRSA colonies and added them to PBS solution for dilution. Then, we added them to the Luria-Bertani (LB) culture medium so that the final concentration of the bacteria was 5 × 10^5^ CFU/mL. We added a higher concentration of the drug and 200 µL bacterial solution mentioned above to the first row of a 96-well plate. We added 100 µL bacterial solution to the remaining row of the 96-well plate without antibiotics. Then, we evenly mixed the bacterial solution containing antibiotics in the first row and took out 100 µL and added into the second row. We mixed the solution in the second row evenly, then extracted 100 µL to the third row, and so on. Then, the 96-well plate was placed in the incubator for 16–20 h, and the absorption at 600 nm was read with a microplate reader. To count the colonies, the cultured bacterial solution was gradually diluted in a gradient of 10 times, dropped on the blood agar plate, and then incubated at 37 ℃ for 18-24 h for bacterial colony count.

#### Staining of live and dead bacteria

DMAO/EthD- III and other dyes were mixed with the bacteria solution to be tested in a certain proportion. They were mixed well and incubated in the dark at room temperature for 15 min. Next, 5-µL stained bacterial suspension droplets were placed on a glass slide with an 18-mm square cover glass and observed by CLSM(Zeiss LSM 800, Germany). Live (green fluorescence) and dead (red fluorescence) bacteria were observed using FITC and CY3 channels, respectively.

### Antibacterial mechanism-related experiments

#### Targeting ability of PLT@Ag-MOF-Vanc observed by SEM

Ag-MOF-Vanc and PLT@Ag-MOF-Vanc were mixed with bacteria for 3 h and fixed at room temperature with 2.5 % glutaraldehyde, 2.5 % paraformaldehyde, and 0.1 M calcium carbonate buffer solution dissolved in deionized water for 2 h. Cover slides were prepared with one drop of 1 % polylysine to capture the cells. We placed one drop of the fixed medium on the cover glass and incubated with water for 5 min before rinsing. The sample was then dehydrated with a series of ethanol concentrations (25 %, 50 %, 75 %, and 95 %) and washed three times in 100 % ethanol. The sample was air-dried for about 24 h, then coated with Au and Pd (7 nm thick) on the biofilm, and observed by SEM (Quanta 250FEG, USA).

#### Targeting ability of PLT@Ag-MOF-Vanc observed by CLSM

The bacteria were co-incubated with DMAO green fluorescent dye for 20 min. After centrifugation, the bacteria solution was resuspended with PBS. Cy5-labeled Ag-MOF-Vanc or Cy5-labeled PLT@Ag-MOF-Vanc were mixed with bacteria for 3 h. After washing with PBS for three times, we collected pictures with CLSM.

#### ATP and F-ATPase

Logarithmic growth MRSA (1 × 10^6^ CFU) was collected and resuspended in PBS buffer (pH = 7.4). Different drugs were added to the bacteria and cultured at 37 °C for 6 h. The bacteria were divided, and the supernatant was collected after centrifugation at 1000 g. Then, the ATP level was determined using the ATP kit (Beyotime Biotechnology, China), and the F-ATPase activity was detected by F-ATPase activity kit (GenMed Scientifics, Shanghai, China).

#### ROS

Logarithmic growth MRSA (1 × 10^6^ CFU) was collected and resuspended in PBS buffer (pH = 7.4). After ROS staining, different drugs were added and incubated at 37 °C for 1 h. Fluorescence was detected by flow cytometry.

#### MDA

Ag-MOF, vancomycin, Ag-MOF-Vanc, or PLT@Ag-MOF-Vanc were co-incubated with bacteria, centrifuged (12,000 rpm, 2 min), resuspended in 1 mL of 2.5 % freshly configured trichloroacetic acid (TCA), and then centrifuged (12,000 rpm, 20 min, 4 °C). The supernatant was diluted with 5 % thiobarbituric acid; the same volume of TCA was added, and the mixture was heated in 100 °C water bath for 30 min, followed by centrifuge (12,000 rpm, 20 min, 4 °C). We determined the absorbance at 532 nm and calculated MDA content (pg/mL) with a molar extinction coefficient of 1.56 nM^− 1^ cm^− 1^.

### Biofilm detection

*CLSM*

100 µL of drug culture solution was added to each well in the fluorescent confocal plate; 100 µl of overnight cultured bacteria solution was inoculated and incubated at 37 °C for 24 h. After washing the plate for three times with PBS, we added DMAO/EthD- III dye and incubated at 37 °C in dark for 20 min. After washing with PBS for three times and drying, we collected pictures with CLSM.

#### Crystal violet staining

We added 100 µl of drug culture solution to each well of the 96-well plate; 100 µl of overnight cultured bacteria solution was inoculated and incubated at 37 °C for 24 h. After washing with PBS for three times, the plate was fixed with methanol. Excess methanol was sucked out; after drying at room temperature, we added 200 µL 1 % crystal violet solution and incubated at room temperature for 15 min. The excess dye was sucked out and washed with PBS for 2–3 times. After drying at room temperature, 200 µl 95 % ethanol was added and incubated for 20 min. The absorbance value was determined at 570 nm.

#### XTT dyeing

XTT with a concentration of 0.2 mg/mL was prepared with PBS, and then the XTT solution was volumized with phenazine methyl ester to a concentration of 0.02 mg/mL. Next, 100 µL of drug culture solution was added to each well of the 96-well plate; 100 µL of overnight cultured bacteria solution was inoculated and incubated at 37 °C for 24 h. After washing the plate with PBS for three times, XTT solution was added and incubated at 37 °C for 3 h, and the absorbance value was determined at 490 nm.

### Mice model of MRSA pneumonia

Twenty-five male Kunming rats at 8 weeks old were randomly divided into five groups: normal saline group, Ag-MOF group, vancomycin group, Ag-MOF-Vanc group and PLT@Ag-MOF-Vanc group. The mice were infected with 150 µL MRSA (concentration 1 × 10^8^ CFU/mL) by tracheal injection. Eight hours after bacterial injection, the mice were given tail vein injection with 100 µL corresponding drugs once a day for 3 consecutive days. HE staining of lung tissue was taken on the first, second, third, and fourth day of treatment to observe the alveolar structure and integrity of ciliated endothelial cells, inflammation, necrosis, and infiltration of inflammatory cells (macrophages) in the alveoli. On day 4, the lung tissues were stained by immunohistochemistry to detect the expression of inflammatory cytokines TNF-α and IL-6. The bacterial count of bronchoalveolar lavage fluid was detected. The whole blood of the mice was collected for blood test indicators (complete blood count, CRP). The levels of TNF-α and IL-6 in blood were detected by ELISA. Another fifty mice with MRSA pneumonia were grouped and treated with corresponding drugs respectively. We recorded the survival status of mice and created the survival curve.

### In vivo imaging

To evaluate the targeting ability of PLT@Ag-MOF-Vanc in vivo, mice model of MRSA pneumonia were injected with Cy5-labeled Ag-MOF-Vanc or Cy5-labeled PLT@Ag-MOF-Vanc through the tail vein. The Xenogen IVIS Lumina XR imaging system (Caliper Life Sciences, USA) was used to evaluate fluorescence signals at 6, 24, and 48 h after administration. After 48 h, the mice were killed by cervical dislocation; the brain, heart, liver, spleen, lung, and kidney were collected. The fluorescence signal was further detected using the Xenogen IVIS Lumina XR imaging system (Caliper Life Sciences, USA).

### In vivo toxicity testing

Healthy male Kunming rats at 8 weeks old were randomized into five groups (n = 5) and injected with 100 µL of normal saline, Ag-MOF, vancomycin, Ag-MOF-Vanc, or PLT@Ag-MOF-Vanc via the tail vein. The mice body weight was measured everyday. One week later, we collected whole blood to test hematological and biochemical indicators (RBC, WBC, PLT, ALT, AST, BUN, CREA). The mice were killed through cervical dislocation. The major organs (heart, liver, spleen, lung, and kidney) were collected, stained with HE, and observed and photographed under microscope.

### Statistical analysis

Data were assessed by GraphPad Prism software and expressed as mean ± SD. Intergroup differences were assessed by oneway ANOVA with subsequent Tukey’s post-test. Significance was demonstrated by p < 0.05.

## Data Availability

All data generated or analysed during this study are included in this published article.
