# Correction to: Platelet membrane-camouflaged silver metal-organic framework drug system against infections caused by methicillin-resistant *Staphylococcus aureus*

**DOI:** 10.1186/s12951-021-01009-w

**Published:** 2021-09-19

**Authors:** Rong Huang, Guang-Qing Cai, Jian Li, Xi-Sheng Li, Hai-Ting Liu, Xue-Ling Shang, Jian-Dang Zhou, Xin-Min Nie, Rong Gui

**Affiliations:** 1 Department of Blood Transfusion, The Third Xiangya Hospital, Central South University, Changsha, Hunan China; 2 Department of Laboratory Medicine, The Third Xiangya Hospital, Central South University, Changsha, Hunan China; 3 Department of Orthopedics, Changsha Hospital of Traditional Chinese Medicine, Changsha Eighth Hospital, Changsha, Hunan China

## Correction to: J Nanobiotechnol (2021) 19:229 https://doi.org/10.1186/s12951-021-00978-2

Following publication of the original article [1], some errors were identified in the original article.

1. The CLSM image of live and dead bacteria for the Ag-MOF-Vanc group after the 16 h treatment had been improperly inserted. The correct image of Fig. 5 and its caption is given in this erratum.

**Fig. 5:**
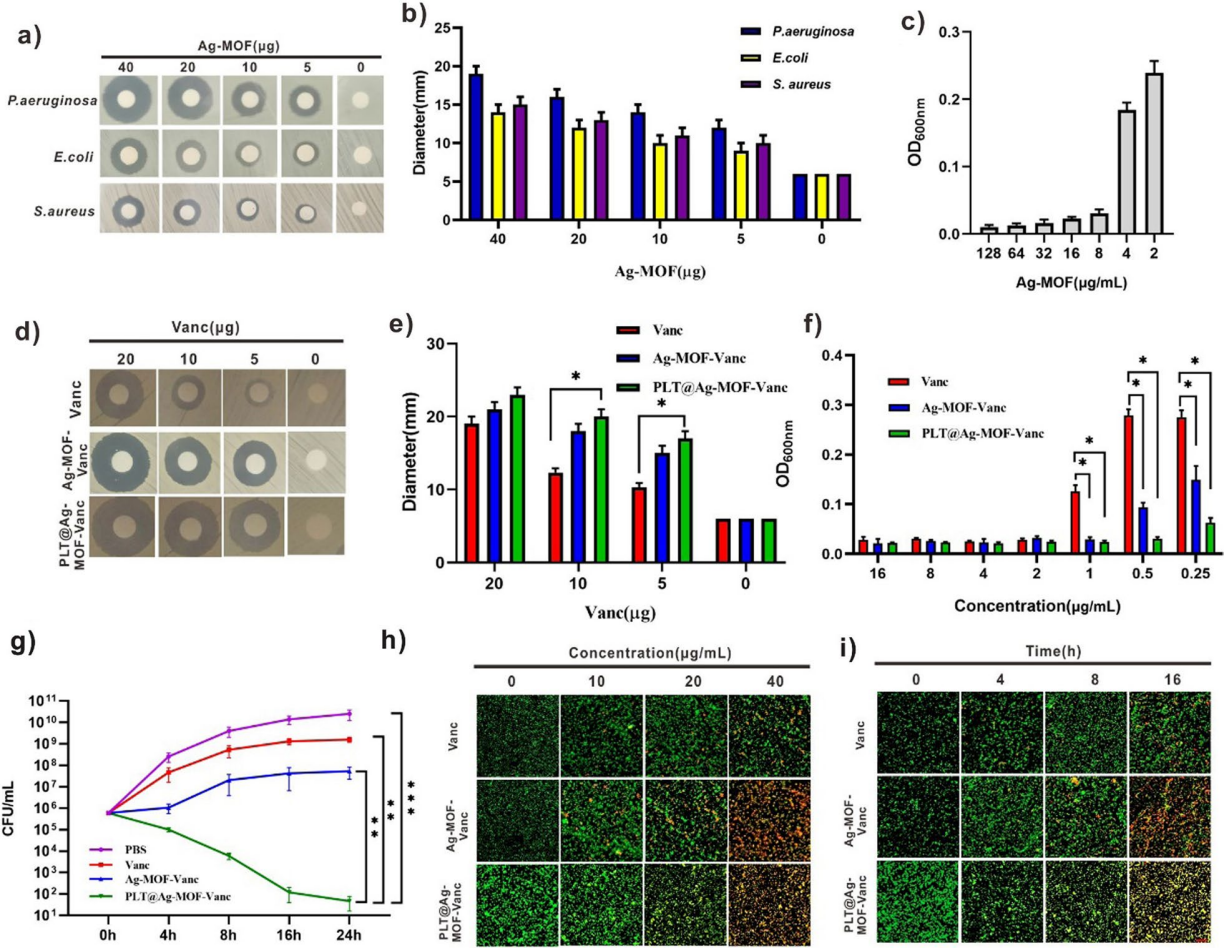
In vitro antibacterial effect of Ag-MOF-Vanc. **a** Inhibition zones and **b** corresponding inhibition zone diameters of Ag-MOF against different bacteria. **c** Concentration effects of Ag-MOF on the growth of MRSA. **d** Inhibition zones, **e** corresponding inhibition zone diameters and **f** concentration effects of Vanc, Ag-MOF-Vanc and PLT@Ag-MOF-Vanc against MRSA. **g** CFU of MRSA treated with 0.5 μg/mL of different drugs. **h** CLSM imaging of death/live staining after exposing MRSA to varying concentrations of Vanc, Ag-MOF-Vanc or PLT@Ag-MOF-Vanc. **i** CLSM imaging of death/live staining after MRSA exposure to Vanc, Ag-MOF-Vanc or PLT@Ag-MOF-Vanc with different incubation time. Scale bar: 20 μm. Data are presented as means ± SD (n = 3).*p < 0.05, **p < 0.01, ***p < 0.001
2. In the paragraphs of “Synthesis of Ag-MOF-Vanc” and “Determination of EE and LE”, the authors miswrote the amount of vancomycin added into synthesis. The correct description is given below:
  We dissolved 0.5 mg Ag-MOF in 1 mL ddH_2_O; **0.4 mg** vancomycin was added, stirred overnight at room temperature by magnetic force, and Ag-MOF-Vanc was obtained by centrifugation.
3. The animal models were established with Kunming mice, and the “Kunming rats” mentioned in some sentences were caused by translation errors.

These errors do not affect the conclusions of this research. The authors apologize for not noticing these errors before publication, and for any inconvenience caused.

The original article has been revised.
